# Low willingness and patient–caregiver concordance regarding long-acting injectable antipsychotics in schizophrenia cases: a convergent mixed-methods study

**DOI:** 10.3389/fpsyt.2026.1931871

**Published:** 2026-08-26

**Authors:** Huaiming Zhang, Bo Wu, Jin Zhou, Pengnan Bai, Yiting Zhu, Yixuan He, Jia Hu, Qi Zhao, Chaowei Fu

**Affiliations:** 1 Department of Community Psychiatry, Mental Health Center Affiliated to Shanghai University of Medicine & Health Sciences, Shanghai, China; 2 Department of Prevention and Treatment, Shanghai Yangpu Mental Health Center, Shanghai, China; 3 School of Public Health, National Health Commission (NHC) Key Laboratory of Health Technology Assessment, Key Laboratory of Public Health Safety, Fudan University, Shanghai, China

**Keywords:** caregivers, long-acting injectable antipsychotics, mixed-methods study, patient and caregiver dyads, schizophrenia

## Abstract

**Introduction:**

Long-acting injectable antipsychotics (LAIAs) may improve treatment continuity in schizophrenia, yet their acceptance in community-based care remains limited. This study examined factors associated with willingness to use LAIAs and dyadic perceptions and barriers among patients with schizophrenia and their primary caregivers in Yangpu District, Shanghai, where LAIA services and financial support were available.

**Methods:**

This convergent mixed-methods study included a paired questionnaire survey of 425 community-managed patients who met LAIA treatment criteria but had not used LAIAs and their primary caregivers. Semi-structured interviews were conducted with 32 patient–caregiver dyads until information saturation was reached. Quantitative and qualitative findings were integrated using a joint display.

**Results:**

Willingness to use LAIAs was low among patients and caregivers (17.4% and 17.6%), and awareness was limited in both groups (26.6% and 28.0%). Four dyadic willingness profiles were identified: concordant unwillingness, patient-only willingness, caregiver-only willingness, and concordant willingness. Higher patient willingness was associated with female sex, patient LAIA awareness, physician recommendation at the highest frequency, and caregiver willingness, whereas higher caregiver willingness was associated with caregiver LAIA awareness, being married, and patient willingness. Qualitative findings explained low acceptance through safety and injection concerns, treatment inertia, limited knowledge, reliance on physicians, family-based decision-making, and service accessibility barriers.

**Discussion:**

These findings suggest that supporting informed LAIA acceptance may require patient–caregiver-centered decision support, safety communication, and coordinated follow-up beyond service availability and cost support.

## 1 Introduction

Schizophrenia is a chronic and disabling mental disorder, with a lifetime prevalence of approximately 1% worldwide (1). In China, an estimated 7 million people live with schizophrenia, and symptom onset commonly occurs in young adulthood (2). Recurrent relapse and long-term impairment in social functioning make sustained treatment and community-based management essential (3–6). The condition also imposes considerable caregiving and financial pressure on families, including an increased risk of catastrophic health expenditure (7).

Long-acting injectable antipsychotics (LAIAs) were developed to support sustained antipsychotic treatment by reducing missed doses and maintaining relatively stable medication exposure over time. Evidence from clinical trials and real-world studies indicated that LAIAs were associated with improved treatment continuity and reduced risks of relapse and rehospitalization among people with schizophrenia (8–11). Despite these potential benefits, LAIAs remained insufficiently implemented in routine community mental health care in China (12). Previous community-based studies had reported limited awareness of LAIAs, low willingness to use LAIAs, and low rates of LAIA prescribing or use among patients with schizophrenia (13, 14).

The limited uptake of LAIAs in community settings may reflect barriers beyond medication availability. Physicians’ willingness to recommend LAIAs and their prescribing preferences may influence whether LAIA treatment is discussed and offered. Clinician-related barriers include concerns about patient acceptance, injection-related issues, and practical implementation, while recent recommendations have emphasized proactive consideration of LAIAs and awareness of potential clinician biases in treatment decision-making (15, 16). Patient-level factors include treatment understanding, perceived benefits, concerns about adverse effects, fear of injections, trust in clinicians, awareness of reimbursement or support policies, and access to injection services (12, 13, 17). Caregivers may further influence LAIA decisions through their involvement in medication supervision and follow-up, as well as through their own knowledge, concerns, caregiving responsibilities, and treatment attitudes (16, 18).

Most previous studies have examined LAIA awareness or acceptance from the perspective of patients, clinicians, or caregivers separately (12, 13). However, LAIA use in community-based schizophrenia care is often embedded in family-based decision-making, where patients’ preferences, caregivers’ concerns, caregiving responsibilities, and follow-up arrangements may interact (18). Evidence remains limited on how willingness to use LAIAs is formed within patient–caregiver dyads, particularly in settings where LAIA services and financial support are already available. Understanding this dyadic decision-making process may help explain why treatment availability does not necessarily translate into acceptance or actual uptake. We conducted a convergent mixed-methods study among patient–caregiver dyads in Yangpu District, where eligible patients could receive LAIA treatment through a designated outpatient service without any out-of-pocket costs after basic medical insurance reimbursement and local financial support (19, 20). We aimed to identify factors associated with current willingness to use LAIAs and to integrate qualitative findings to explain dyadic perceptions and barriers to LAIA uptake in community-based care.

## 2 Materials and methods

### 2.1 Study design, setting and participants

We conducted a convergent mixed-methods study in 2025 in Yangpu District, Shanghai, combining a paired quantitative survey with qualitative in-depth interviews among patients diagnosed with schizophrenia according to ICD-10 criteria who were registered in the local community-based management system for serious mental disorders (21, 22), met the eligibility criteria for LAIA treatment, and had not yet used LAIAs, together with their primary caregivers.

Patients were included if they met all of the following criteria: (1) registered in Yangpu District and receiving community-based service management; (2) identified through comprehensive community risk assessment as either having a risk of serious behavioral incidents, or experiencing illness instability related to poor treatment adherence (missed or refused oral medication during follow-up in the previous year), weak family supervision, or absence of family supervision; (3) assessed by qualified mental health professionals as clinically suitable for LAIA treatment; (4) clinically stable, with no acute episode in the previous 6 months; (5) aged 18–65 years; and (6) provided informed consent and voluntarily agreed to participate in the study. Patients were excluded if they met any of the following criteria: (1) currently receiving LAIA treatment; (2) previously used LAIAs but discontinued treatment; (3) had severe cognitive impairment that prevented completion of the questionnaire or interview; (4) currently in an acute episode; or (5) had severe physical illness that could affect treatment decision-making.

Primary caregivers were defined as family members or legal guardians who were involved in daily care, treatment supervision, follow-up arrangements, or treatment decision-making. Caregivers were included if they met all of the following criteria: (1) being the primary caregiver or legal guardian of the patient; (2) aged 18 years or older; and (3) provision of informed consent and voluntary agreement to participate. The study protocol was approved by the Ethics Committee of Shanghai Yangpu District Mental Health Center (approval number: 202412). Written informed consent was obtained from all participating patients and caregivers.

### 2.2 Sampling

For the quantitative survey, stratified random sampling was used to recruit eligible patient–caregiver dyads from the community-based management system for serious mental disorders, covering all community management areas in Yangpu District. The sample size was calculated using an expected LAIA willingness rate of 10.0%, a margin of error of 0.03, and a two-sided Z value of 1.96. The required sample size was 385 dyads. A total of 425 valid patient–caregiver dyads were included in the quantitative analysis.

For the qualitative component, patient–caregiver dyads were randomly selected from the quantitative survey participants and interviewed until information saturation was reached, defined as the point at which no new major themes emerged in consecutive interviews. A total of 32 patient–caregiver dyads were interviewed.

### 2.3 Data collection and measures

A structured paired questionnaire was administered separately to patients and corresponding caregivers. Face and content validity were assessed by relevant experts, and the questionnaire was pilot-tested before formal administration, with an overall Cronbach’s α of 0.875, subscale α coefficients of 0.736–0.913, and a test–retest reliability coefficient of 0.83. The questionnaire collected sociodemographic characteristics, awareness of LAIAs, current willingness regarding LAIA use, reasons for willingness or unwillingness, and potential triggers for choosing LAIA treatment using a multiple-response item from both members of each dyad. Physician recommendation was assessed by the frequency of recommendations per month. Patient-specific items included disease- and treatment-related characteristics, community risk assessment level, current medication status, and perceived adverse-effect severity. Caregiver-specific items included caregiver–patient relationship and willingness for the patient to use LAIAs. Patient and caregiver questionnaires were linked using unique dyad identifiers. Completed questionnaires were checked on site for completeness and consistency, and data were double-entered using EpiData 3.1.

The primary outcome was current willingness to use LAIAs, assessed as patients’ willingness to use LAIAs and caregivers’ willingness for the patient to use LAIAs. Responses were classified as willing or unwilling. LAIA awareness was defined as the ability to identify at least one specific LAIA with the generic or brand name, such as paliperidone palmitate, fluphenazine decanoate, or haloperidol decanoate (23). Adverse-effect severity was assessed using a self-reported five-point rating scale for side effects of current antipsychotic medication, ranging from 1 = no obvious side effects to 5 = severe side effects substantially affecting normal daily life. Higher scores indicated greater perceived adverse-effect severity.

For the qualitative component, semi-structured individual in-depth interviews were conducted separately with patients and caregivers. The interview guide focused on sources of LAIA awareness, the formation of willingness to use LAIAs, perceived difficulties and challenges in LAIA promotion, and suggestions for improving LAIA implementation. All interviews were audio-recorded with permission and transcribed into text within 24 hours after completion.

### 2.4 Data analysis and integration

Categorical variables were described as frequencies and percentages, and continuous variables were described as means and standard deviations. Differences in characteristics according to current willingness to use LAIAs were compared using the chi-square test or Fisher’s exact test. Binary logistic regression models were used to identify factors associated with current willingness to use LAIAs among patients and caregivers. Dyads were classified into four willingness groups based on patient and caregiver responses: concordant unwillingness, patient-only willingness, caregiver-only willingness, and concordant willingness. Multinomial logistic regression was used to examine factors associated with these dyadic willingness patterns. Results were presented as odds ratios (ORs) or adjusted odds ratios (aORs) with 95% confidence intervals (CIs). All quantitative analyses were conducted using SPSS version 27.0, with a two-sided P value <0.05 considered statistically significant.

Qualitative data were analyzed using thematic analysis. Patient and caregiver transcripts were coded separately and then compared to identify common and group-specific themes related to LAIA willingness, concerns, and decision-making.

Quantitative and qualitative findings were integrated at the interpretation stage using a joint display. Survey and regression findings were compared with interview-based explanations to develop mixed-methods inferences on LAIA awareness, patient–caregiver influence, safety concerns, family decision-making, and implementation barriers.

## 3 Results

### 3.1 Participant characteristics

Among the 425 patient–caregiver dyads, 74 patients (17.4%) and 75 caregivers (17.6%) expressed current willingness to use LAIAs. Overall, 52.9% of patients were female and the mean age was 55.94 ± 16.65 years, 48.9% had more than 9 years of education, and 26.6% were aware of LAIAs. Significant differences between willingness groups were observed for sex, age, and LAIA awareness.

Among caregivers, 50.4% were female and the mean age was 59.95 ± 8.32 years, 44.7% had more than 9 years of education, and 28.0% were aware of LAIAs. LAIA awareness was higher among willing than unwilling caregivers (52.0% vs 22.9%, P<0.001), whereas other sociodemographic characteristics were broadly comparable (**Table 1**).

**Table 1:** Characteristics of patients and caregivers according to willingness to use long-acting injectable antipsychotics.

Characteristics	Patients				Caregivers			
	Overall (N = 425)	Willing (N = 74)	Unwilling (N = 351)	P value	Overall (N = 425)	Willing (N = 75)	Unwilling (N = 350)	P value
Male	200 (47.0)	27 (36.5)	173 (49.3)	0.045	211 (49.6)	36 (48.0)	175 (50.0)	0.753
Age, years	55.94 ± 16.65	53.84 ± 14.60	56.34 ± 16.99	0.300	59.95 ± 8.32	59.14 ± 9.16	60.12 ± 8.13	0.355
≥ 60 years	377 (88.7)	59 (79.7)	318 (90.6)	0.007	388 (91.3)	66 (88.0)	322 (92.0)	0.265
Education > 9 years	208 (48.9)	38 (51.4)	170 (48.4)	0.648	190 (44.7)	31 (41.3)	159 (45.4)	0.517
Married	198 (46.6)	34 (45.9)	164 (46.7)	0.903	230 (54.1)	47 (62.7)	183 (52.3)	0.102
Monthly household income per capita, CNY				0.206				0.617
≤ 3000	72 (16.9)	16 (21.6)	56 (16.0)		55 (12.9)	8 (10.7)	47 (13.4)	
3001–5000	193 (45.4)	27 (36.5)	166 (47.3)		206 (48.5)	40 (53.3)	166 (47.4)	
≥ 5001	160 (37.6)	31 (41.9)	129 (36.8)		164 (38.6)	27 (36.0)	137 (39.1)	
Awareness of LAIAs	113 (26.6)	35 (47.3)	78 (22.2)	<0.001	119 (28.0)	39 (52.0)	80 (22.9)	<0.001
Medical payment method				0.220				0.604
Employee insurance	218 (51.3)	38 (51.4)	180 (51.3)		253 (59.5)	46 (61.3)	207 (59.1)	
Resident insurance	175 (41.2)	27 (36.5)	148 (42.2)		151 (35.5)	27 (36.0)	124 (35.4)	
Other	32 (7.5)	9 (12.2)	23 (6.6)		21 (4.9)	2 (2.7)	19 (5.4)	
Primary caregiver relationship				0.299				0.574
Spouse	101 (23.8)	14 (18.9)	87 (24.8)		101 (23.8)	18 (24.0)	83 (23.7)	
Parents	164 (38.6)	36 (48.6)	128 (36.5)		164 (38.6)	32 (42.7)	132 (37.7)	
Other	160 (37.6)	24 (32.5)	136 (38.7)		160 (37.6)	25 (33.0)	135 (38.6)	
Disease duration, years	27.34 ± 14.34	25.38 ± 13.29	27.75 ± 14.53	0.173	–	–	–	–
Family history of mental illness	50 (11.8)	12 (16.2)	38 (10.8)	0.191	–	–	–	–
Current medication status				0.221	–	–	–	–
No medication	65 (15.3)	7 (9.5)	58 (16.5)		–	–	–	
Monotherapy	193 (45.4)	33 (44.6)	160 (45.6)		–	–	–	
Combination therapy	167 (39.3)	34 (45.9)	133 (37.9)		–	–	–	
Adverse effects	2.93 ± 1.42	2.59 ± 1.37	3.00 ± 1.42	0.393	–	–	–	–

### 3.2 Dyadic willingness profiles

The 425 dyads were classified into four willingness profiles: concordant unwillingness (P−/C−, n=311, 73.2%), patient-only willingness (P+/C−, n=39, 9.2%), caregiver-only willingness (P−/C+, n=40, 9.4%), and concordant willingness (P+/C+, n=35, 8.2%). The P−/C− group had the highest proportion of dyads in which neither patients nor primary caregivers were aware of LAIAs (67.8%), whereas the P+/C+ group had the highest proportion of dyads in which both were aware of LAIAs (40.0%). The P+/C− group had lower proportions of patients and caregivers aged ≥60 years, while disease duration was longest in the P−/C+ group and shortest in the P+/C+ group. Current medication status also differed across the four profiles (**Table 2**). Patient sex, patient education, perceived adverse-effect severity, primary caregiver education, and relationship to the patient did not differ significantly across groups.

**Table 2:** Characteristics of patient–primary caregiver dyads according to dyadic willingness profiles for LAIAs.

Characteristics	P−/C− (n=311)	P+/C− (n=39)	P−/C+ (n=40)	P+/C+ (n=35)	P value
Dyadic awareness pattern					<0.001
Neither aware of LAIAs, n (%)	211 (67.8)	16 (41.0)	14 (35.0)	12 (34.3)	
Patient-only awareness, n (%)	32 (10.3)	11 (28.2)	6 (15.0)	4 (11.4)	
Primary caregiver-only awareness, n (%)	32 (10.3)	6 (15.4)	16 (40.0)	5 (14.3)	
Both aware of LAIAs, n (%)	36 (11.6)	6 (15.4)	4 (10.0)	14 (40.0)	
Patient characteristics					
Male, n (%)	151 (48.6)	13 (33.3)	23 (57.5)	14 (40.0)	0.129
Age, years	55.86 ± 16.93	54.90 ± 16.70	60.27 ± 17.29	52.58 ± 11.81	0.294
≥60 years, n (%)	283 (91.0)	29 (74.4)	35 (87.5)	30 (85.7)	0.018
Education >9 years, n (%)	152 (48.9)	23 (59.0)	18 (45.0)	15 (42.9)	0.505
Disease duration, years, mean ± SD	26.26 ± 14.14	22.68 ± 14.58	35.29 ± 17.89	19.62 ± 11.87	<0.001
Current medication status, n (%)					0.028
No medication	51 (16.4)	5 (12.8)	7 (17.5)	2 (5.7)	
Monotherapy	134 (43.1)	14 (35.9)	26 (65.0)	19 (54.3)	
Combination therapy	126 (40.5)	20 (51.3)	7 (17.5)	14 (40.0)	
Primary caregiver characteristics					
Age ≥60 years, n (%)	289 (92.9)	31 (79.5)	38 (95.0)	30 (85.7%)	0.015
Education >9 years, n (%)	141 (45.3)	18 (46.2)	13 (32.5)	18 (51.4)	0.371
Relationship to patient, n (%)					0.053
Spouse	74 (23.8)	9 (23.1)	13 (32.5)	5 (14.3)	
Parents	118 (37.9)	14 (35.9)	10 (25.0)	22 (62.9)	
Other caregivers	119 (38.3)	16 (41.0)	17 (42.5)	8 (22.9)	

P−/C− indicates patient unwilling/primary caregiver unwilling; P+/C−, patient willing/primary caregiver unwilling; P−/C+, patient unwilling/primary caregiver willing; and P+/C+, patient willing/primary caregiver willing. Values are presented as n (%) unless otherwise indicated. LAIAs, long-acting injectable antipsychotics.

### 3.3 Factors associated with individual and dyadic willingness to use LAIAs

In the adjusted logistic regression model for patients, caregiver willingness showed the strongest association with patient willingness to use LAIAs (aOR=8.00, 95% CI: 4.25–15.09). Patient LAIA awareness was also associated with higher willingness (aOR=2.50, 95% CI: 1.37–4.56). Female sex was associated with higher patient willingness (aOR=2.01, 95% CI: 1.10–3.63). The highest physician recommendation frequency was significantly associated with patient willingness overall (P = 0.037).

For caregivers, patient willingness was strongly associated with caregiver willingness regarding LAIA use (aOR=7.69, 95% CI: 4.06–14.58). Caregiver awareness of LAIAs (aOR=4.08, 95% CI: 2.25–7.39) and being married (aOR=2.08, 95% CI: 1.12–3.85) were also associated with higher caregiver willingness. Physician recommendation frequency was not significantly associated with caregiver willingness (all P>0.05). Other sociodemographic characteristics and primary caregiver relationship were not significantly associated with caregiver willingness.

**Figure 1:**
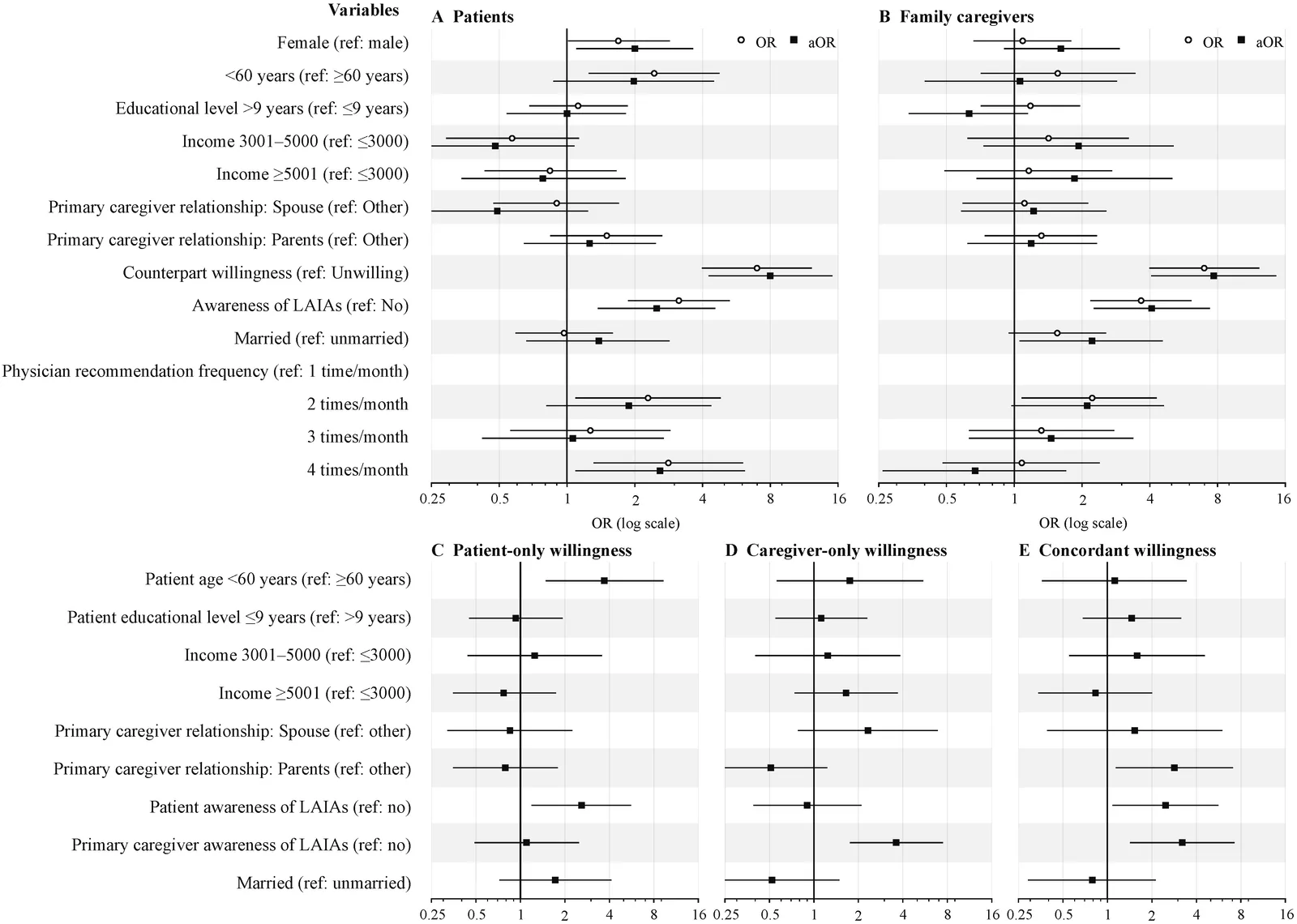
Factors associated with patient, caregiver, and dyadic willingness to use long-acting injectable antipsychotics. Panels **(A, B)** show unadjusted and adjusted logistic regression results, and panels **(C–E)** show adjusted multinomial logistic regression results with concordant unwillingness as the reference. Estimates are ORs with 95% CIs on a log scale.. LAIAs, long-acting injectable antipsychotics.

In the multinomial logistic regression model of dyadic willingness patterns, concordant unwillingness was used as the reference outcome. Patient-only willingness was associated with younger patient age (aOR=3.70, 95% CI: 1.48–9.29) and patient LAIA awareness (aOR=2.59, 95% CI: 1.19–5.62). Caregiver-only willingness was mainly associated with caregiver awareness of LAIAs (aOR=3.60, 95% CI: 1.75–7.46). Concordant willingness was associated with parental caregiver relationship compared with other family caregivers (aOR=2.84, 95% CI: 1.14–7.06), patient awareness of LAIAs (aOR=2.48, 95% CI: 1.08–5.65), and caregiver awareness of LAIAs (aOR=3.21, 95% CI: 1.42–7.25). Overall, LAIA awareness and patient–caregiver mutual willingness appeared more closely associated with willingness patterns than socioeconomic characteristics (**Figure 1**).

### 3.4 Patient–caregiver perspectives on willingness to use LAIAs

A total of 32 patient–caregiver dyads participated in the qualitative interviews. The sociodemographic and clinical characteristics of the interviewed patients and caregivers are summarized in **Supplementary Tables S1** and **S2**. The qualitative interviews identified five integrated themes related to current willingness to use LAIAs: safety and injection concerns, convenience and treatment inertia, limited knowledge and professional reliance, family role and shared decision-making, and service accessibility. Patients and caregivers both expressed concerns about adverse effects and injections. Patients tended to emphasize immediate discomfort, injection pain, and uncertainty about changing from current oral medication, whereas caregivers focused more on long-term safety, reversibility, adverse-effect management, and the practical burden of arranging repeated visits. Both groups recognized the potential advantages of LAIAs, including reduced daily medication burden and financial support. At the same time, limited knowledge, reliance on physician explanations and guidance, family-based decision-making, and access barriers continued to affect acceptance and implementation (**Table 3**). Detailed coding frameworks and representative quotations are provided in **Supplementary Tables S3** and **S4**.

**Table 3:** Integrated qualitative themes from patient–caregiver interviews on willingness to use long-acting injectable antipsychotics.

Integrated theme	Patient perspective	Caregiver perspective	Main interpretation
Safety and injection concerns	*“I’m really scared of injections. That’s a hurdle I just can’t easily get over.”*	*“Once the shot is given, it lasts for a whole month. If it doesn’t suit him, that whole month will be hard.”*	Safety concerns were shared, but patients emphasized immediate discomfort whereas caregivers emphasized controllability.
Convenience and treatment inertia	*“The pills I take now aren’t perfect, but at least I know how they work, and my body’s already used to them.”*	*“I wouldn’t have to watch him take pills every day. That would really take a load off me.”*	Convenience and financial support promoted willingness, but treatment inertia limited acceptance.
Limited knowledge and professional reliance	*“If the doctor explains it clearly, how it works, whether it helps, and what side effects there might be, I’d think about trying it.”*	*“I don’t really know much about long-acting injectable antipsychotics. I’ve only heard the name.”*	Low medication literacy made physician communication central to decision-making.
Family role and shared decision-making	*“If my family supports me getting the injection, and the doctor also says it’s suitable, I’d consider it.”*	*“I don’t really support it. The medicine he’s taking now seems okay, so I don’t tend to change it.”*	LAIA acceptance reflected family-based decision-making.
Service accessibility and follow-up burden	*“The inconvenient part is that I have to go to the hospital for the shot, and it’s only available on Wednesday mornings. If I can’t make it, I have to wait until the next week.”*	*“If he needs the injection, I’d have to take time off or rearrange my schedule.”*	Access barriers and follow-up burden could limit actual uptake.

Quotes are illustrative English translations of participant interviews and were edited slightly for readability while preserving the original meaning. LAIAs, long-acting injectable antipsychotics. Themes were derived from thematic analysis of in-depth interviews with 32 patient–caregiver dyads. The table summarizes integrated theme domains based on patient-derived and caregiver-derived qualitative findings.

### 3.5 Integration of quantitative and qualitative findings

The integration of quantitative and qualitative findings showed both convergence and complementarity. The strong association between patient and caregiver willingness in the quantitative analyses was supported by interview findings, which indicated that LAIA decisions were rarely made by patients alone and were often shaped by family judgement, caregiving responsibility, and daily treatment supervision. The positive association between LAIA awareness and willingness was further contextualized by qualitative evidence showing that many participants had limited knowledge of LAIAs and relied heavily on physicians for explanations of treatment benefits, risks, and follow-up requirements. Qualitative interviews further clarified that concerns about side effects, injection pain, and perceived irreversibility were central barriers to acceptance. In the survey, physician recommendation was the most frequently selected potential trigger for considering LAIA use, reported by 143 patients and 142 caregivers in the descriptive multiple-response analysis. In addition, qualitative findings extended the quantitative results by highlighting clear physician communication and guidance, policy clarity, and service accessibility as practical conditions relevant to informed consideration of LAIA treatment. These findings suggest that LAIA acceptance in community-based care was shaped not only by patient-level awareness, but also by family-based decision-making, physician communication and guidance, and the feasibility of routine injection services (**Table 4**).

**Table 4:** Joint display integrating quantitative and qualitative findings on current willingness to use long-acting injectable antipsychotics among patient–caregiver dyads.

Integrated domain	Quantitative signal	Qualitative explanation	Mixed-methods inference
Patient–caregiver dyadic influence	Patient and caregiver willingness were strongly associated with each other.	Decisions were rarely made by patients alone and often depended on caregiver judgement and support.	LAIA acceptance was embedded in dyadic, family-based decision-making.
Medication awareness and treatment literacy	Awareness of LAIAs was associated with higher willingness in both groups.	Participants often lacked detailed knowledge about drug options, injection frequency, benefits, risks, and follow-up.	Awareness was associated with willingness, but informed acceptance required more detailed medication counselling.
Safety and adverse-effect concerns	Safety and adverse-effect concerns were mainly identified through qualitative interviews.	Patients and caregivers worried about adverse effects, injection fear, and perceived irreversibility.	Safety concerns centred on monitoring, adverse-effect management, and perceived reversibility.
Caregiver-role heterogeneity	Parental caregiver relationship was associated with concordant willingness compared with other caregivers.	Parents, spouses, and other caregivers differed in caregiving burden and decision logic.	Caregiver relationship may shape decision logic and perceived responsibility.
Physician recommendation and professional guidance	Physician recommendation was the most frequently selected positive trigger and was associated with higher patient willingness at the highest recommendation frequency.	Participants emphasized the need for clear physician explanations of treatment benefits, potential adverse effects, and follow-up requirements.	Clear physician communication and guidance may help translate general awareness of LAIAs into informed treatment consideration.
Cost coverage and policy trust	Income and insurance type were not consistently associated with willingness.	Zero-cost treatment was valued, but unclear policy details and safety concerns remained.	Cost coverage was valued, but uncertainty about policy details and treatment safety remained.
Service accessibility and implementation barriers	Accessibility was mainly identified through qualitative interviews.	Barriers included injection location, travel distance, clinic schedules, and repeated visits.	Service arrangements could influence whether willingness translated into actual LAIA use.

## 4 Discussion

This convergent mixed-methods study showed that willingness to use LAIAs remained low among community-managed patients with schizophrenia and their primary caregivers, despite the availability of designated LAIA services and local financial support. This finding highlights an implementation gap between treatment availability and treatment acceptance. Integrated findings suggest that LAIA acceptance was shaped by treatment literacy, perceived safety and controllability, patient–caregiver decision-making, and the feasibility of regular injection visits in routine community care.

The low level of willingness observed in this study was consistent with previous community-based studies in China (12–14), which reported limited awareness, acceptance, and use of LAIAs among patients with schizophrenia. However, our study extends previous evidence by examining willingness within patient–caregiver dyads and within a local context where economic support and a designated LAIA service pathway were already in place. The persistence of low willingness in this context suggests that expanding service availability and reducing out-of-pocket costs, while necessary, may be insufficient to generate informed acceptance without structured communication and follow-up support. The higher willingness observed among female patients should be interpreted cautiously. Previous studies suggest that preferences for LAIAs may be shaped by prior treatment experiences, perceptions of treatment convenience and control, injection-related concerns, and family or social support (17, 24). The association with being married among caregivers may similarly reflect differences in caregiving context and available support rather than marital status itself, as social support has been shown to influence caregiving experiences among families of people with schizophrenia (25). These potential mechanisms were not directly assessed in the present study.

An important implication of the integrated findings was that LAIA decision-making in community-based schizophrenia care should not be conceptualized solely as an individual patient choice (15). This interpretation is consistent with previous studies emphasizing shared decision-making and family involvement in long-term schizophrenia care (26, 27). In our study, patient and caregiver willingness were strongly associated, while the qualitative findings indicated that caregivers were involved in treatment appraisal, risk interpretation, medication supervision, follow-up coordination, and judgements about whether LAIA treatment was compatible with routine family care. Together, these findings suggest that LAIA consideration often occurred within a patient–caregiver context. This interpretation is consistent with previous evidence that caregivers contribute to clinician communication (27), adherence support (28), and relapse-related monitoring in schizophrenia care (18). However, as actual LAIA initiation or refusal was not assessed, concordance in willingness should not be interpreted as evidence that caregiver attitudes directly determined treatment uptake; shared information exposure and other unmeasured dyad-level factors may also have contributed to this association.

Our findings indicated that LAIA acceptance depended on treatment-specific understanding and confidence in treatment safety. Although LAIA awareness was associated with greater willingness in both patients and caregivers (12, 13, 29), the qualitative findings suggested that awareness alone was insufficient for informed acceptance. Patients and caregivers expressed concerns about expected treatment effects (30), injection-related discomfort (13), adverse-effect management (17), and whether LAIA treatment could be adjusted after initiation (17, 30). These concerns were consistent with previous studies reporting that limited knowledge (13), concerns about adverse-effect management (30), fear of injection, and perceived loss of treatment control may hinder LAIA acceptance (13, 31). Accordingly, community-based LAIA services should emphasize physician-led education and counselling on treatment benefits, potential adverse effects, and treatment adjustability, while involving both patients and caregivers in decision-making and follow-up planning.

The subgroup findings further suggested that patterns of LAIA willingness may differ across parent, spouse, and other caregiver groups, potentially reflecting heterogeneity in caregiving roles, decision authority, and perceived responsibility for long-term illness management. In parent-caregiver dyads, LAIA decisions may have been closely linked to concerns about relapse prevention, daily supervision, and the long-term sustainability of caregiving, reflecting the long-term role of parents in caring for adult children with serious mental illness (32, 33). In dyads involving spouses as primary caregivers, LAIA decisions may have been shaped more by shared living arrangements, mutual negotiation, and concerns about the effects of treatment on daily family life. This interpretation is consistent with evidence that spousal caregiving in schizophrenia is closely related to family functioning, caregiving burden, and marital role expectations (34). For patients supported by other caregivers, willingness may have depended more on the availability of practical support and the clarity of follow-up responsibilities, highlighting the need to clarify caregiving roles and service coordination in community-based LAIA implementation.

The predominance of older participants was not attributable to age-based sampling, as the age distribution of our sample was generally consistent with that of the LAIA-eligible population under community-based management in the study area. This suggests that the age structure of the sample largely reflected that of the underlying eligible population. Nevertheless, the findings should be generalized cautiously to younger patients with schizophrenia and their caregivers, who were less represented in the eligible population.

This study had several strengths. First, the patient–caregiver dyad design allowed us to examine LAIA willingness as a shared treatment decision rather than as an isolated patient or caregiver attitude. Second, the convergent mixed-methods design enabled integration of quantitative associations with qualitative explanations, clarifying the perceptions and decision-making processes underlying low willingness despite financial support and treatment availability. Third, the study was conducted in a community-based management setting with designated LAIA services and local financial protection, which strengthened its relevance for real-world implementation. Several limitations should be noted. First, the cross-sectional design precludes causal inference, and residual confounding and reverse causality cannot be excluded. Second, the study was conducted in Yangpu District, Shanghai, where designated LAIA services and local financial support were already available; therefore, the limited role of economic factors in this setting may not apply to areas with weaker service capacity or less comprehensive reimbursement support. Third, although eligible patients were aged 18–65 years, the study sample was predominantly composed of older adults. This may partly reflect the relatively older age structure of the local population under long-term community management, but younger patients remained underrepresented in our sample. The findings should therefore be generalized cautiously to younger patients with schizophrenia and their caregivers. Fourth, LAIA awareness, and current willingness were self-reported and may have been affected by recall bias, reporting bias, or social desirability bias.

Willingness to use LAIAs remained low among community-managed patients with schizophrenia and their caregivers despite treatment availability and financial support in this study population. LAIA acceptance was shaped by treatment literacy, safety confidence, patient–caregiver decision-making, and the feasibility of integrating LAIAs into routine community care. Future policy efforts should therefore build on service access and financial protection by strengthening structured decision support, safety communication, and follow-up coordination in routine community-based LAIA care.

## Data Availability

The raw data supporting the conclusions of this article will be made available by the authors, without undue reservation.
