# Supplementary material for: Low willingness and patient–caregiver concordance regarding long-acting injectable antipsychotics in schizophrenia cases: a convergent mixed-methods study
Source: Front Psychiatry. 2026 Aug 26;17:1931871. doi: 10.3389/fpsyt.2026.1931871 (PMC13553541; doi:10.3389/fpsyt.2026.1931871)
Supplement: Supplementary file 1 [file Table1.docx]

**Supplementary Table S1 Sociodemographic and clinical characteristics of interviewed patients**

| ID | Sex | Age, years | Disease duration,  years | Medication  adherence | Monthly household  income, CNY | Insurance type |
| --- | --- | --- | --- | --- | --- | --- |
| P1 | Male | 33 | 6 | Regular | 5001–10000 | Resident insurance |
| P2 | Male | 26 | 5 | Regular | 5001–10000 | Resident insurance |
| P3 | Male | 60 | 9 | Irregular | ≤3000 | Resident insurance |
| P4 | Female | 25 | 8 | Irregular | 3001–5000 | Employee insurance |
| P5 | Male | 46 | 6 | Irregular | ≤3000 | Resident insurance |
| P6 | Female | 41 | 4 | Regular | 3001–5000 | Employee insurance |
| P7 | Female | 48 | 21 | Regular | ≤3000 | Resident insurance |
| P8 | Male | 43 | 5 | Regular | 3001–5000 | Resident insurance |
| P9 | Female | 48 | 8 | Irregular | ≤3000 | Resident insurance |
| P10 | Female | 29 | 22 | Regular | ≤3000 | Resident insurance |
| P11 | Female | 42 | 23 | Regular | 5001–10000 | Resident insurance |
| P12 | Male | 39 | 4 | Irregular | 3001–5000 | Employee insurance |
| P13 | Male | 45 | 9 | Irregular | 5001–10000 | Resident insurance |
| P14 | Male | 41 | 7 | Irregular | 5001–10000 | Resident insurance |
| P15 | Female | 50 | 14 | Regular | 5001–10000 | Resident insurance |
| P16 | Male | 28 | 6 | Regular | 5001–10000 | Resident insurance |
| P17 | Male | 49 | 15 | Irregular | 5001–10000 | Resident insurance |
| P18 | Male | 32 | 24 | Irregular | 5001–10000 | Resident insurance |
| P19 | Male | 43 | 16 | Irregular | ≤3000 | Resident insurance |
| P20 | Female | 57 | 8 | Regular | 5001–10000 | Resident insurance |
| P21 | Male | 34 | 14 | Regular | 5001–10000 | Resident insurance |
| P22 | Female | 26 | 6 | Irregular | ≤3000 | Employee insurance |
| P23 | Male | 30 | 5 | Regular | 5001–10000 | Employee insurance |
| P24 | Male | 55 | 20 | Irregular | 5001–10000 | Resident insurance |
| P25 | Female | 38 | 20 | Irregular | 3001–5000 | Resident insurance |
| P26 | Female | 53 | 19 | Regular | ≤3000 | Employee insurance |
| P27 | Male | 46 | 3 | Regular | 5001–10000 | Employee insurance |
| P28 | Male | 29 | 25 | Regular | ≤3000 | Employee insurance |
| P29 | Female | 29 | 19 | Irregular | 5001–10000 | Resident insurance |
| P30 | Male | 59 | 7 | Irregular | ≤3000 | Resident insurance |
| P31 | Female | 37 | 6 | Irregular | 3001–5000 | Resident insurance |
| P32 | Female | 54 | 4 | Regular | 3001–5000 | Resident insurance |

**Supplementary Table S2 Characteristics of interviewed caregivers and their corresponding patients**

| ID | Relationship to patient | Patient sex | Patient age, years | Duration of illness, years | Medication  adherence | Monthly household income, CNY | Insurance type |
| --- | --- | --- | --- | --- | --- | --- | --- |
| F1 | Sibling | Male | 33 | 6 | Regular | 5001–10000 | Resident insurance |
| F2 | Spouse | Male | 26 | 5 | Regular | 5001–10000 | Resident insurance |
| F3 | Parent | Male | 60 | 9 | Irregular | ≤3000 | Resident insurance |
| F4 | Parent | Female | 25 | 8 | Irregular | 3001–5000 | Employee insurance |
| F5 | Parent | Male | 46 | 6 | Irregular | ≤3000 | Resident insurance |
| F6 | Parent | Female | 41 | 4 | Regular | 3001–5000 | Employee insurance |
| F7 | Child | Female | 48 | 21 | Regular | ≤3000 | Resident insurance |
| F8 | Spouse | Male | 43 | 5 | Regular | 3001–5000 | Resident insurance |
| F9 | Child | Female | 48 | 8 | Irregular | ≤3000 | Resident insurance |
| F10 | Parent | Female | 29 | 22 | Regular | ≤3000 | Resident insurance |
| F11 | Spouse | Female | 42 | 23 | Regular | 5001–10000 | Resident insurance |
| F12 | Parent | Male | 39 | 4 | Irregular | 3001–5000 | Employee insurance |
| F13 | Parent | Male | 45 | 9 | Irregular | 5001–10000 | Resident insurance |
| F14 | Parent | Male | 41 | 7 | Irregular | 5001–10000 | Resident insurance |
| F15 | Child | Female | 50 | 14 | Regular | 5001–10000 | Resident insurance |
| F16 | Child | Male | 28 | 6 | Regular | 5001–10000 | Resident insurance |
| F17 | Sibling | Male | 49 | 15 | Irregular | 5001–10000 | Resident insurance |
| F18 | Spouse | Male | 32 | 24 | Irregular | 5001–10000 | Resident insurance |
| F19 | Parent | Male | 43 | 16 | Irregular | ≤3000 | Resident insurance |
| F20 | Parent | Female | 57 | 8 | Regular | 5001–10000 | Resident insurance |
| F21 | Sibling | Male | 34 | 14 | Regular | 5001–10000 | Resident insurance |
| F22 | Spouse | Female | 26 | 6 | Irregular | ≤3000 | Employee insurance |
| F23 | Parent | Male | 30 | 5 | Regular | 5001–10000 | Employee insurance |
| F24 | Child | Male | 55 | 20 | Irregular | 5001–10000 | Resident insurance |
| F25 | Parent | Female | 38 | 20 | Irregular | 3001–5000 | Resident insurance |
| F26 | Spouse | Female | 53 | 19 | Regular | ≤3000 | Employee insurance |
| F27 | Spouse | Male | 46 | 3 | Regular | 5001–10000 | Employee insurance |
| F28 | Parent | Male | 29 | 25 | Regular | ≤3000 | Employee insurance |
| F29 | Child | Female | 29 | 19 | Irregular | 5001–10000 | Resident insurance |
| F30 | Sibling | Male | 59 | 7 | Irregular | ≤3000 | Resident insurance |
| F31 | Parent | Female | 37 | 6 | Irregular | 3001–5000 | Resident insurance |
| F32 | Parent | Female | 54 | 4 | Regular | 3001–5000 | Resident insurance |

**Supplementary Table S3 Qualitative coding framework for patient and caregiver interviews**

| Interview group | Theme | Subtheme | Selected initial codes |
| --- | --- | --- | --- |
| Patients | Concerns about adverse effects and injection fear | Fear of adverse effects | Concerns that adverse effects may be stronger than those of oral medication; concerns about symptom fluctuation |
|  |  | Concerns about the perceived irreversibility of injections | Concern that the medication cannot be withdrawn once injected |
|  |  | Fear of injection itself | Fear of injection pain; perception that injections are frightening |
|  | Convenience needs and zero-cost incentives | Burden of medication management | Daily oral medication is inconvenient; frequently forgetting to take medication |
|  |  | Appeal of financial incentives | Acceptance of the zero-cost policy |
|  |  | Reliance on the current treatment regimen | Current oral medication is perceived as acceptable; reluctance to change treatment |
|  | Limited medication knowledge and passive decision-making | Limited knowledge of LAIAs | Lack of understanding of long-acting injectable antipsychotics |
|  |  | Dependence on physician recommendation | Reliance on doctors’ recommendations |
|  |  | Influence of family support | Decisions influenced by whether family members support LAIA use |
|  | Service accessibility constraints | Time and geographic barriers | Inconvenient clinic hours; long travel distance to healthcare facilities |
| Caregivers | Concerns about adverse effects and safety | Fear of irreversible adverse effects | Concerns that adverse effects may be irreversible; perception that changing medication is risky |
|  |  | Fear of injection | Concerns about injection pain |
|  |  | Long-term safety concerns | Concerns about long-term safety |
|  | Zero-cost policy incentives and trust issues | Appeal of financial incentives | Acceptance of the zero-cost policy |
|  |  | Unclear policy information | Lack of understanding of policy details |
|  | Limited medication knowledge and decision dependence | Limited knowledge of LAIAs | Lack of understanding of long-acting injectable antipsychotics |
|  |  | Dependence on physician recommendation | Reliance on doctors’ recommendations |
|  | Caregiver role and differentiated decision logic | Caregiving burden | Burden of supervising daily medication; concerns about missed doses |
|  |  | Preference for maintaining the current regimen | Current oral medication is perceived as acceptable; reluctance to change treatment |
|  | Service accessibility barriers | Time and geographic barriers | Inconvenient clinic hours; long travel distance to healthcare facilities |

Note: LAIAs, long-acting injectable antipsychotics. This table presents the coding framework derived from thematic analysis of patient and caregiver interviews. Patient and caregiver interviews were coded separately and then summarized into themes and subthemes.

**Supplementary Table S4 Representative quotations supporting qualitative themes**

| Integrated theme | Interview group | Representative quotation |
| --- | --- | --- |
| Safety and injection-related concerns | Patient | “What if the adverse effects are serious?” |
|  | Patient | “Once the injection is given, it cannot be taken back.” |
|  | Patient | “Will the injection hurt?” |
|  | Caregiver | “Unlike oral medication, which can be stopped if it causes discomfort, an injection cannot be taken back once given.” |
|  | Caregiver | “A long-acting injection lasts for a month. If the patient does not tolerate it well, that whole month could be difficult.” |
|  | Caregiver | “Will it affect liver or kidney function? Will it cause dependence?” |
| Convenience needs and treatment inertia | Patient | “Taking medication every day is troublesome, and I often forget.” |
|  | Patient | “The current medication is working reasonably well, so I do not want to change it.” |
|  | Caregiver | “Supervising daily medication is tiring.” |
|  | Caregiver | “I worry when doses are frequently missed.” |
| Cost coverage and policy trust | Patient | “The policy is attractive because patients do not need to pay out of pocket.” |
|  | Caregiver | “I am not clear about the details of the policy.” |
| Limited medication knowledge and decision dependence | Patient | “I do not know much about long-acting injections.” |
|  | Patient | “If the doctor strongly recommends it, I would consider it.” |
|  | Caregiver | “I do not really understand long-acting medication.” |
|  | Caregiver | “I would rely on the doctor’s recommendation.” |
| Family support and caregiver-role differences | Patient | “If my family supports me in using long-acting injections, I would consider it.” |
|  | Caregiver | “We know how the current medication works, and the patient has adapted to it. If we change to a long-acting injection, we do not know whether it will work.” |
| Service accessibility barriers | Patient | “The injection is available only on Wednesday mornings.” |
|  | Patient | “Where I live is a bit far from the district mental health center.” |
|  | Caregiver | “The clinic time is inconvenient.” |
|  | Caregiver | “The hospital is far away.” |

**Note:** LAIAs, long-acting injectable antipsychotics. Quotations were translated from Chinese and lightly edited for clarity. The table presents representative quotations supporting the qualitative themes identified from patient and caregiver interviews.
